# Complete Assembly of Escherichia coli Sequence Type 131 Genomes Using Long Reads Demonstrates Antibiotic Resistance Gene Variation within Diverse Plasmid and Chromosomal Contexts

**DOI:** 10.1128/mSphere.00130-19

**Published:** 2019-05-08

**Authors:** Arun Gonzales Decano, Catherine Ludden, Theresa Feltwell, Kim Judge, Julian Parkhill, Tim Downing

**Affiliations:** a School of Biotechnology, Dublin City University, Dublin, Ireland; b Wellcome Trust Sanger Institute, Hinxton, United Kingdom; c London School of Hygiene & Tropical Medicine, London, United Kingdom; U.S. Centers for Disease Control and Prevention

**Keywords:** MGE, antibiotic resistance, genome assembly, plasmid, sequencing

## Abstract

The incidence of infections caused by extraintestinal Escherichia coli (ExPEC) is rising globally, which is a major public health concern. ExPEC strains that are resistant to antimicrobials have been associated with excess mortality, prolonged hospital stays, and higher health care costs. E. coli sequence type 131 (ST131) is a major ExPEC clonal group worldwide, with variable plasmid composition, and has an array of genes enabling antimicrobial resistance (AMR). ST131 isolates frequently encode the AMR genes *bla*_CTX-M-14_, *bla*_CTX-M-15_, and *bla*_CTX-M-27_, which are often rearranged, amplified, and translocated by mobile genetic elements (MGEs). Short DNA reads do not fully resolve the architecture of repetitive elements on plasmids to allow MGE structures encoding *bla*_CTX-M_ genes to be fully determined. Here, we performed long-read sequencing to decipher the genome structures of six E. coli ST131 isolates from six patients. Most long-read assemblies generated entire chromosomes and plasmids as single contigs, in contrast to more fragmented assemblies created with short reads alone. The long-read assemblies highlighted diverse accessory genomes with *bla*_CTX-M-15_, *bla*_CTX-M-14_, and *bla*_CTX-M-27_ genes identified in three, one, and one isolates, respectively. One sample had no *bla*_CTX-M_ gene. Two samples had chromosomal *bla*_CTX-M-14_ and *bla*_CTX-M-15_ genes, and the latter was at three distinct locations, likely transposed by the adjacent MGEs: IS*Ecp1*, IS*903B,* and Tn*2*. This study showed that AMR genes exist in multiple different chromosomal and plasmid contexts, even between closely related isolates within a clonal group such as E. coli ST131.

**IMPORTANCE** Drug-resistant bacteria are a major cause of illness worldwide, and a specific subtype called Escherichia coli ST131 causes a significant number of these infections. ST131 bacteria become resistant to treatments by modifying their DNA and by transferring genes among one another via large packages of genes called plasmids, like a game of pass-the-parcel. Tackling infections more effectively requires a better understanding of what plasmids are being exchanged and their exact contents. To achieve this, we applied new high-resolution DNA sequencing technology to six ST131 samples from infected patients and compared the output to that of an existing approach. A combination of methods shows that drug resistance genes on plasmids are highly mobile because they can jump into ST131’s chromosomes. We found that the plasmids are very elastic and undergo extensive rearrangements even in closely related samples. This application of DNA sequencing technologies illustrates at a new level the highly dynamic nature of ST131 genomes.

## Abstract (precis)

Drug-resistant bacteria are a major cause of illness worldwide, and a specific subtype called Escherichia coli ST131 causes a significant number of these infections. ST131 bacteria become resistant to treatments by modifying their DNA and by transferring genes among one another via large packages of genes called plasmids, like a game of pass-the-parcel. Tackling infections more effectively requires a better understanding of what plasmids are being exchanged and their exact contents. To achieve this, we applied new high-resolution DNA sequencing technology to six ST131 samples from infected patients and compared the output to that of an existing approach. A combination of methods shows that drug resistance genes on plasmids are highly mobile because they can jump into ST131’s chromosomes. We found that the plasmids are very elastic and undergo extensive rearrangements even in closely related samples. This application of DNA sequencing technologies illustrates at a new level the highly dynamic nature of ST131 genomes.

## INTRODUCTION

Reported cases of bloodstream and urinary tract infections caused by extraintestinal pathogenic Escherichia coli (ExPEC) are increasing globally at an alarming rate (1). As a key source of ExPEC isolates worldwide, E. coli sequence type 131 (ST131) is regarded as a serious threat to public health, given its high level of antimicrobial resistance (AMR), as well as the broad spectrum of infections it causes in community and hospital settings (2, 3).

E. coli ST131 is virulent (4) and has an expansive range of virulence factors (5, 6), especially those linked to uropathogenic E. coli (UPEC) (3, 7, 8). AMR and virulence genes allow ST131 to adapt to drug selection pressure and to survive in extraintestinal niches and are often encoded on mobile genetic elements (MGEs) (9), which means that the exact set of virulence and AMR genes in a single ST131 isolate may vary (8, 10). ST131 encodes a range of extended-spectrum β-lactamases (ESBLs) that hydrolyze third-line drugs, including cephalosporins, the most common of which is cefotaximase *bla*_CTX-M-15_ . Within ST131, clade C2 has more AMR genes than other clades and is typically *bla*_CTX-M-15_ positive, differentiating it from clade C1, which can be *bla*_CTX-M-14_ or *bla*_CTX-M-27_ positive (3, 8).

Most ST131 AMR genes are reported to be carried on plasmids, circular self-replicating double-stranded DNA molecules that constitute part of the bacterial accessory genome (11–13). Plasmids can reduce bacterial cell fitness, but a number of postsegregation killing and stable plasmid inheritance mechanisms allow the stable maintenance of IncF plasmids in ST131 (14–16). The chromosomal integration of plasmid genes is most commonly facilitated by transposons, which can ensure the acquisition and conservation of such elements if there is no subsequent local recombination (17, 18).

Identifying plasmid conjugation, recombination, and transposition could have value in tracking AMR genes associated with disease outbreaks and antibiotic treatment failures. Plasmids may be classified using incompatibility (Inc), relaxase (MOB), and mating pair formation system typing (19), but difficulties in plasmid genetic analysis and reconstruction arise with short-read data due to rearrangements driven by recombination, dense arrays of repetitive elements including transposable elements (TEs), changes in gene copy numbers, and high sequence variation. Methods using short reads alone may fail to detect genomic segments exchanged between plasmids and the chromosome, limiting evaluation of the core and accessory genomes.

Whole-genome sequencing has provided a high resolution of the genomic epidemiology of ST131 and plasmid-mediated AMR outbreaks (20). However, short reads alone are insufficient to resolve plasmids that often have numerous small MGEs of ∼1 kb or less in size, e.g., TEs and insertion sequences (ISs) (21). Complex transposable units (TUs) consisting of multiple TEs or ISs can mobilize AMR genes by transposition, and this can sometimes be followed by recombination within the TU between one of the inverted repeats (IRs) flanking the TE and the IR of another local TE or an adjacent homologous sequence, resulting in different TU structures, locations, and copy numbers. At present, the exact resolution of complex structural rearrangements of repetitive TUs containing AMR genes may be impossible with short reads (22). Consequently, plasmid assembly is a challenge, requiring accurate long reads and sufficient coverage to distinguish between independent plasmids with regions of sequence identity (21, 23).

Long reads, such as those generated using Oxford Nanopore Technologies (ONT) or Pacific Biosciences platforms, can provide a solution to this plasmid assembly problem (24–26). Here, we sequenced six ST131 using the ONT GridION X5 platform. Using the resulting high-coverage sequence data, we reconstructed and annotated the plasmids and chromosomal regions carrying *bla*_CTX-M_ genes, as well as their genetic context and copy numbers.

## RESULTS

### Oxford Nanopore long-read quality control and filtering.

High-molecular-weight DNA from six E. coli ST131 isolates was sequenced using long Oxford Nanopore reads and short Illumina reads to assemble their genomes, allowing for plasmid reconstruction and resolution of AMR genes, MGEs, and associated rearrangements. The ONT GridION X5 sequencing generated 8.9 Gbases in total across 1,406,087 reads (mean length of 6.3 kb) (Table 1). The number of reads generated per hour, total yield of bases over time, read length distribution, and read Q score distribution were examined (see Fig. S2 at https://ndownloader.figshare.com/files/14983688). Half of the bases with Q values of ≥7 were on reads of 18 kb or longer (Fig. S3). These metrics indicated sufficient GridION data in terms of quantity and quality. Initial screening removed reads with Q values of <7, leaving 1,142,067 reads with 8.2 Gbases with a mean Q score of 10.2 and a mean length of 7.2 kb (Table 1) for analysis. This included 81 reads longer than 100 kb, including one of 155,312 bases. This corresponded to a 257-fold theoretical coverage for six 5.3-Mb genomes.

**TABLE 1:** Quality parameters indicate high-quality read libraries for the six ST131 samples from GridION X5 sequence data^*a*^

Parameter	Value for:	
	All reads	Reads with a Q of ≥7
Total bases	8,908,946	8,193,921
Total reads	1,406,087	1,142,067
Mean length (bp)	6,336	7,175
Median length (bp)	2,273	2,897
Mean Q score	9.1	10.2
Median Q score	10.0	10.5
Reads of >100 kb	85	81

a A total of 264,020 low-quality reads (with a Q of <7) totaling 715,024,800 bases were excluded.

The initial number of reads per library ranged from 127,118 to 510,253, and these were filtered using a series of steps to ensure that the reads used for each of the six assemblies had high quality. Bases were successfully called at an average of 97.9% of reads (Table 2). Identifying the consensus demultiplexed, duplicate-free, and adapter-free reads from Porechop v0.2.4 eliminated a further 2.9% of the base-called reads, yielding 120,123 to 487,482 reads per library (Table 2).

**TABLE 2:** Number of reads generated from GridION X5 sequencing data per library that passed filtering during base calling with Albacore v2.0 and those that were adapter-free (using Porechop v0.2.4)^*a*^

Strain	No. of initial reads (fast5)	No. of base-called reads (fastq)	No. of adapter-free reads (fastq)	Avg length (bp)
VRES1160	358,829	351,636	345,033	7,037
VREC0693	208,478	204,904	194,413	8,982
VRES0739	163,349	160,693	155,900	9,171
VREC1013	510,253	497,646	487,482	6,657
VREC1073	313,627	304,218	298,658	7,256
VREC1428	127,118	124,539	120,123	9,301

a The adapter-free reads totaling 1,601,609 were used for downstream analyses. A total of 80,045 reads were excluded during base calling or adapter trimming.

### Long-read genome assembly illuminates highly diverse accessory genomes.

All six genome assemblies produced chromosomes of 4.81 to 5.38 Mb, with differing numbers of plasmids with lengths spanning 4 to 156 kb (see Fig. S4 at https://ndownloader.figshare.com/files/14983688; Table 3). The numbers of contigs produced by long-read assemblies of two samples (VREC0693, VRES0739) corresponded exactly to the chromosome and plasmids. The others had either one (VREC1073, VRES1160, VREC1013) or two (VREC1428) additional chromosomal contigs (Table S2).

**TABLE 3:** Total sizes of assemblies, chromosomes, and plasmids found in each strain based on their optimal whole-genome assemblies using the GridION X5 long reads^*a*^

Strain	Genome length (bp)	No. of contigs		*N*_50_	Chromosome size (Mb)	No. of plasmids	Plasmid size(s) (kb)
		Assembled	Minimum possible				
VRES1160	5,326,801	6	5	5,126,679	5.23	4	62, 16, 5, 4
VREC0693	5,260,741	3	3	5,039,909	5.04	2	132, 89
VRES0739	4,806,912	3	3	4,797,749	4.81	2	5, 4
VREC1013	5,223,433	3	2	3,699,451	5.14	1	90
VREC1073	5,539,158	3	2	5,286,804	5.38	1	156
VREC1428	5,236,419	7	5	4,924,536	5.13	4	92, 5, 5, 4

a Each assembly had seven or fewer contigs, and in three cases, no fewer contigs were possible, consistent with full genome assembly (for VREC0693, VRES0739, and VREC1073). The optimal assembly with Unicycler used long reads alone (in bold mode), with the exception of VREC1013, for which a hybrid combining short Illumina reads with long Oxford Nanopore reads was best, with minor manual screening (see supplemental results at https://ndownloader.figshare.com/files/14983688).

Contigs were classified as chromosomal or plasmid derived using mlplasmids given a probability threshold of 60% (22), with further screening for plasmid-related gene content using the Multiple Antibiotic Resistance Annotator (MARA), the Comprehensive Antibiotic Resistance Database (CARD), and PlasmidFinder (Table S2). The largest plasmid was a 156.3-kb IncFIA plasmid in VREC1073, its sole plasmid. VREC1428 and VRES1160 had 92.8- and 61.9-kb IncFIA plasmids, respectively, along with three small Col plasmids each (Table 3). VREC0693 had a 132.0-kb IncFIB plasmid and an 88.8-kb IncB plasmid; IncB plasmids have the same Rep domains as IncFII plasmids (27). VREC3013 had one 89.9-kb IncFII plasmid. VRES0739 alone had no large plasmid, which was verified with the short-read data.

By mapping the long reads to the optimal assemblies, the read coverage of each chromosome and plasmid was estimated (see Table S2 at https://ndownloader.figshare.com/files/14983688). Each chromosome had 126- to 310-fold median coverage, and the median coverage levels of large plasmids ranged from 85- to 282-fold, except for VREC1013’s IncFII plasmid, which had 1,015-fold coverage and a normalized depth of 3.3-fold. The normalized depth of plasmids compared to chromosomes suggested that some cells in VREC1428 and VREC1073 may have lost their IncFIA plasmid, and the same was true for VREC0693 and its IncFIB plasmid. However, the IncFIA plasmid in VRES1160 and the IncB plasmid in VREC0693 had higher than expected copy numbers (by 9% after normalization), potentially indicating stable plasmid retention.

Across five assemblies in the Unicycler normal mode, the median insertion/deletion (indel) error rates for short reads and hybrid assemblies were similar (0.21 and 0.28 per 100 kb, respectively) but were much higher for long-read assemblies (265.0 per 100 kb) (Table S3). Likewise, the median mismatch error rates for short reads and hybrid assemblies were comparable (4.25 and 2.28 per 100 kb, respectively), but the error rate was much higher for long-read assemblies (332.8 per 100 kb) (Table S3). These rates excluded VREC1073, for which some Quast metrics were zero values. Similarly, the recovery of conserved BUSCO genes was far higher for hybrid assemblies (>99.5%) than for long-read ones (>82.3%).

### The dynamic locations and genomic contexts of *bla*_CTX-M_ genes in long-read assemblies.

**FIG 1:**
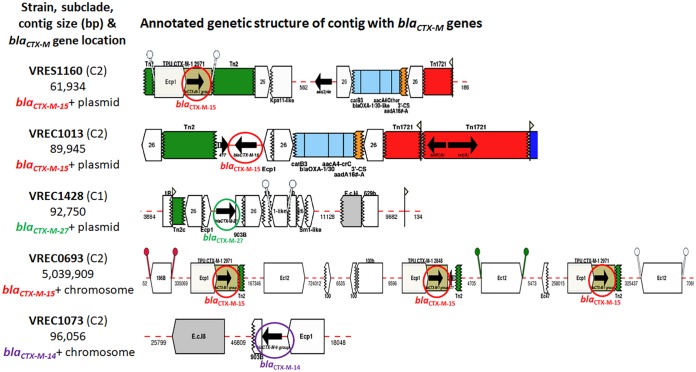
The structure of contigs with *bla*_CTX-M_ genes. Two of the ST131 *bla*_CTX-M_ genes are on chromosomal contigs (VREC0693 and VREC1073). VRES1160 and VREC1013 have IncFIA and IncFII plasmids, respectively, both of which have *bla*_CTX-M-15_ genes. VREC1428 has an IncFIA plasmid with the *bla*_CTX-M-27_ gene. VRES0739 is not shown because it was *bla*_CTX-M_ negative and had no large plasmid. The contigs were classified as chromosomal or plasmid derived by mlplasmids so that the *bla*_CTX-M_ genes and their genetic flanking context could be examined. Annotation was derived from GalileoAMR based on the Multiple Antibiotic Resistance Annotator (MARA) and database. The *bla*_CTX-M_ variants are labeled and circled in red (*bla*_CTX-M-15_), purple (*bla*_CTX-M-14_), or green (*bla*_CTX-M-27_).

The optimized assemblies provided an improved view of the genomic context of each *bla*_CTX-M_ allele, whose effectiveness as a marker for ST131 clade classification and origin (8) were explored in this study. The deeper resolution of genome architecture revealed surprising differences in *bla*_CTX-M_ gene context (Fig. 1; see Table S2 at https://ndownloader.figshare.com/files/14983688), including the discovery of chromosomal *bla*_CTX-M_ genes in VREC0693 (three copies of *bla*_CTX-M-15_) and VREC1073 (one copy of *bla*_CTX-M-14_). All *bla*_CTX-M_ genes were complete (876 bp) with adjacent IS*Ecp1* (1,658 bp with flanking IRs of 14 to 16 bp) and Tn*2* (5.8-kb) elements; IS*Ecp1* and Tn*2* can transpose *bla*_CTX-M_ and other ESBL genes (28, 29). The VRES0739 genome did not contain any region homologous to *bla*_CTX-M_, most likely because it had lost an IncF plasmid, unlike the other isolates.

VRES1160, VREC0693, and VREC1013 all had *bla*_CTX-M-15_ genes linked to isoforms of IS*Ecp1*, IS*26,* and Tn*2*, implicating them in driving the transposition of the TU (Fig. S5). Each was similar to the ST131 clade C2 *ISEcp1-bla_CTX_*_-M-15_-orf477Δ TU (8, 30) but with distinct structural differences. VRES1160’s single *bla*_CTX-M-15_ gene was at bp 2296 on its IncFIA plasmid and was flanked by IS*Ecp1* to its 5′ end and by Tn*2* followed by IS*26* at its 3′ end, with another Tn*2* 5′ of IS*Ecp1*. VREC0693’s three chromosomal *bla*_CTX-M-15_ genes were not tandem repeats (chromosomal locations at positions 2781074 bp, 3696068 bp, and 3970927 bp), but each of these TUs were identical: all had IS*Ecp1* at the 5′ ends and truncated Tn*2*’s at the 3′ ends. VREC1013’s sole *bla*_CTX-M-15_ gene was located at bp 13226 on its IncFII plasmid and was flanked by a truncated IS*Ecp1* at its 5′ end and Tn*2* at its 3′ end, with IS*26* copies 5′ and 3′ of these segments.

VREC1428’s single *bla*_CTX-M-27_ gene was on its IncFIA plasmid at position 6018, and VREC1073’s single chromosomal *bla*_CTX-M-14_ gene started at contig position 19746 (see Fig. S5 at https://ndownloader.figshare.com/files/14983688). Both their *bla*_CTX-M_ genes were flanked by a truncated IS*Ecp1* at the 5′ ends and a shortened IS*903B* at the 3′ ends, suggesting that IS*Ecp1* and IS*903B* may have facilitated the transposition of the TU from the plasmid. Similar *bla*_CTX-M_ gene transposition events have been observed in ST131 clade C1 (8).

**FIG 2:**
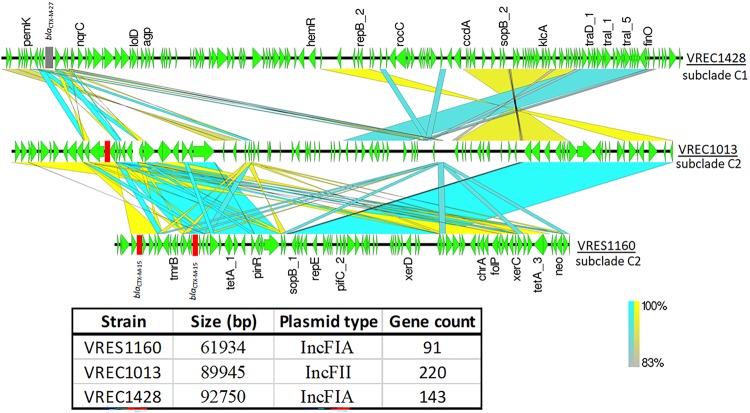
Pairwise comparisons of the three *bla*_CTX-M_-positive plasmid-associated contigs show high sequence identity for the two from subclade C2 (VREC1013 and VRES1160) relative to that of one from C1 (VREC1428, top). The BLAST result was visualized with EasyFig v2.2.2 such that the blocks connecting the regions of the contigs represent nucleotide homology: blue for homologous regions in the same direction, and yellow for inversions. Gaps or white spaces denote unique loci or regions present in one contig but not in the other. Gene models are in green, with the direction of transcription shown by arrows. Genes of interest are labeled above each arrow. The *bla*_CTX-M-27_ gene (top) is in mauve, and the two *bla*_CTX-M-15_ genes (middle and bottom) are in red. The table at the bottom shows the contig size, plasmid type, and number of genes per strain. The list and products of the annotated genes are given in Table 5.

Alignment of the plasmid-derived contigs of VRES1160 (IncFIA) to VREC1013 (IncFIB) showed that the *bla*_CTX-M-15_-positive plasmids were much more similar (>83% identity) than VREC1428’s *bla*_CTX-M-27_-positive IncFIA plasmid, which was more distinct (Fig. 2). In addition, VREC1428’s plasmid had *traI* and *traD* genes, indicating conjugation machinery (Table 4), as well as high homology to at least one published plasmid, unlike VRES1160’s and VREC1013’s plasmids (see supplemental results at https://ndownloader.figshare.com/files/14983688). This suggested that the VRES1160 and VREC1013 plasmids had homology corresponding well with *bla*_CTX-M_ gene and subclade classification and that they were structurally different from published plasmids due to recombination.

**TABLE 4:** Genes in the plasmid contigs of VREC1013, VRES1160, and VREC1428^*a*^

Genes in plasmid contigs of indicated isolate		
VREC1428	VREC1013	VRES1160
*pemI*	*xerD_1*	***bla_1***
*pemK*	*ccdA*	*tmrB*
***bla***	*vapC_1*	*cat*
*nqrC*	*vapC_2*	***bla_2***
*lolD*	*kdgT_1*	*aacA4*
*agp*	*kdgT_2*	*tetA_1*
*hemR*	*ridA*	*tetA_2*
*repB_1*	*yagE*	*pinR*
*repB_2*	*ugpA*	*sopB_1*
*mmuM*	*cpdA*	*sopB_2*
*rocC*	*tnpA*	*repE*
*ccdA*	*yknY*	*ccdB*
*ccdB*	*tpd*	*ccdA*
*sopB_1*	*xerD_2*	*pifC_1*
*sopB_2*	*dhfrI_1*	*pifC_2*
*klcA*	*ant1_1*	*pifC_3*
***traD_1***	*folP*	*repB_1*
***traD_2***	*srpC*	*repB_2*
***traD_3***	***bla***	*xerD*
***traD_4***	*xerD_3*	*chrA*
***traD_5***	*xerC*	*folP*
***traD_6***	*dhfrI_2*	*mdtJ*
***traI_1***	*ant1_2*	*xerC*
***traI_2***	*umuC*	*tetA_3*
***traI_3***	*lexA*	*tetR*
***traI_4***	*klcA*	*neo*
***traI_5***		*tnpR*
***traI_6***		
***traI_7***		
*finO*		

a Count numbers are indicated by an underline followed by the number of counts (e.g., *_1*). The *bla*_CTX-M_ (*bla*), *traI,* and *traD* genes are in bold. Only isolate VREC1428 had *traI* and *traD* genes, indicating conjugative capacity. VREC0693, VRES0739, and VREC1073 contigs did not have *tra* genes.

**TABLE 5:** Protein products encoded by genes found in plasmids of strains VREC1013, VRES1160, and VREC1428^*a*^

Gene	Protein product
*agp*	Glucose-1-phosphatase
*ccDA*	Antitoxin (plasmid maintenance)
*chrA*	Response regulator
*finO*	Fertility inhibition protein
*folP*	Dihydropteroate synthase
*hemR*	Hemin TonB-dependent receptor
*klcA*	Antirestriction protein
*lolD*	Lipoprotein-releasing system ATP-binding
*neo*	Aminoglycoside 3′-phosphotransferase
*nqrC*	Na(+)-translocating NADH-quinone reductase
*pemK*	mRNA interferase
*pifC*	Transcriptional repressor protein
*pinR*	Serine recombinase protein
*repB*	Replication protein
*repE*	Replication initiation protein
*rocC*	Amino acid permease
*sopB*	Inositol phosphate phosphatase
*tetA*	Tetracycline resistance protein
*tmrB*	Tunicamycin resistance protein
*tnpR*	Transposon gamma-delta resolvase
*traD*	Coupling protein
*traI*	Multifunctional conjugation protein
*xerC*	Tyrosine recombinase protein
*xerD*	Tyrosine recombinase protein

a See Fig. 2.

### Phylogenetic context of analyzed isolates.

**FIG 3:**
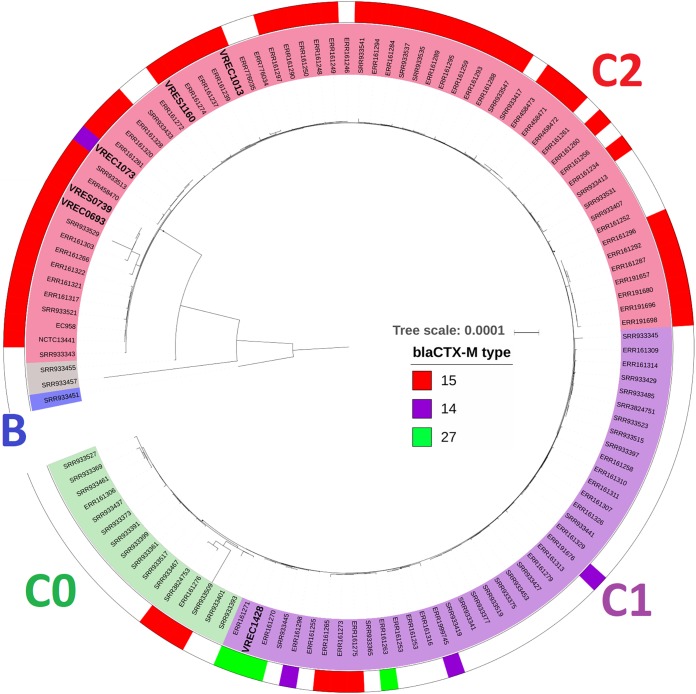
The phylogenetic context of the six ST131 genomes (names in large bold font) shows that all except that of VREC1428 are in ST131 subclade C2 (red inner ring, VRES1160, VREC1073, VRES0739, VREC0693, and VREC1013). VREC1428 clustered in subclade C1 (purple inner ring). No new isolate clustered in C0 (green inner ring), B (blue inner ring), or an intermediate cluster (gray inner ring). Clade classification was based on phylogenetic analysis (8) by including the reference NCTC13441, 63 isolates from reference 8, and 56 isolates from reference 31 with associated classification and *bla*_CTX-M_ allele data. VREC1073 and VREC0693 had chromosomal *bla*_CTX-M_ genes. The outer ring shows *bla*_CTX-M-15_ (red), *bla*_CTX-M-14_ (purple), and *bla*_CTX-M-27_ alleles (green). The phylogeny was built with RAxML v8.2.11 using 4,457 SNPs from a core genome alignment generated with Roary v3.11.2 and was visualized with iTOL v4.3. Branch support was performed by 100 bootstrap replicates, and the scale bar indicates the number of substitutions per site. This midpoint-rooted phylogeny includes reference genome isolates EC958 and NCTC13441 (both in C2).

A comparison of these six samples with 119 published ST131 isolates (8, 31) as short-read assemblies scaffolded using reference genome NCTC13441 showed that all clustered in ST131 clade C (Fig. S6). There was sufficient resolution across 4,457 core genome single-nucleotide polymorphisms (SNPs) to confidently assign them to subclade C1 (*n* = 1) or C2 (*n* = 5) (Fig. 3). VRES1160, VREC0693, VREC1013, VRES0739, and VREC1073 clustered with C2, whereas the *bla*_CTX-M-27_-positive VREC1428 was in C1. VRES1160, VREC0693, and VREC1013 all had IncF plasmids (IncFIA, IncFIB, IncFII) and *bla*_CTX-M-15_ genes, consistent with C2 isolates, which are typically *bla*_CTX-M-15_ positive; 77% of the C2 isolates here (48 out of 62) were observed to be *bla*_CTX-M-15_ positive. However, VREC1073 was in subclade C2 but had a *bla*_CTX-M-14_ gene, contradicting this pattern, and was the sole *bla*_CTX-M-14_-positive C2 isolate found here. The core genomes of VRES0739 and VREC0693 were identical, implying that VRES0739 has very recently lost its (*bla*_CTX-M_-positive IncF) plasmid. The sole isolate clustering with C1 was VREC1428, which had an IncFIA plasmid with a *bla*_CTX-M-27_ gene and so may belong to the emerging subclade C1-M27, as evidenced by the presence of prophage-like regions like M27PP1/2 (31).

## DISCUSSION

Our study resolved the plasmid architecture of several recent E. coli ST131 isolates, allowing investigation of AMR gene location, copy number, and potential transposon-driven rearrangements. This advance was facilitated by careful DNA handling during extraction to produce large volumes of high-molecular-weight DNA that was pure and free from contamination, which was avoided by performing separate extraction steps to obtain small plasmids (32), overcoming a limitation for MinION sequencing (21).

The long-read genome assemblies illuminated significant variations in plasmids, MGEs, and *bla*_CTX-M_ gene composition that were not captured by short reads. ST131 is a globally pandemic E. coli clonal group (15) with diverse sources of transmission (25). Phylogenetic comparison with published genomes (8, 31) showed that five out of six isolates were from subclade C2, with one from C1. The emergence of clade C has been associated with IncF plasmids, and subclade C2 has been associated with IS*Ecp1* and Tn*2* elements flanking *bla*_CTX-M-15_ genes (33, 34). Our long-read assemblies showed the excision of the entire TU from the IncFIB plasmid and chromosomal integration at three distinct locations for VREC0693 and, similarly, chromosomal translocation of the *bla*_CTX-M-14_ gene from an IncFIA plasmid for VREC1073, mediated by IS*Ecp1* and IS*903B* based on previous work (8). These transposition events were likely driven by recombination at adjacent transposable elements. This highlights the value of long-read sequencing to resolve the location of *bla*_CTX-M_ genes and that chromosomal translocations are not rare in ST131.

A high resolution of the AMR gene structure, context, and copy number is highly predictive of AMR phenotypes (35) and could lead to new insights into AMR mechanisms. However, the high indel and mismatch errors in long Oxford Nanopore reads (32, 36–38) limit the power to identify AMR isoforms that could permit genome-based antimicrobial susceptibility testing (27, 39, 40). Here, the five ONT assemblies together had an average of 447-fold higher indel and 48-fold higher mismatch error rates than those for the corresponding Illumina reads, similar to previous work with MinION reads (23), and this impacted gene identification. Consequently, short reads and assembly polishing methods remain important for SNP identification and error detection until long-read error rates can be reduced (41).

Our findings illustrate the diversity of AMR gene context even within recently emerged clones such as ExPEC ST131. The detection of multiple instances of chromosomally integrated ESBL genes using long reads here for *bla*_CTX-M-15_ in E. coli has parallels elsewhere for *bla*_OXA-181_ in *bla*_CTX-M-15_-positive Klebsiella pneumoniae (42) and so highlights chromosomal ESBL gene IS*Ecp1*-mediated transposition as a potential adaptive mechanism in Enterobacteriaceae. Further studies with larger sample sizes are needed to identify the rates and mechanisms of these dynamic changes.

## MATERIALS AND METHODS

### Sample collection.

Six ESBL-producing E. coli ST131 clinical strains were isolated in June to October 2015 from patients at Addenbrooke’s Hospital, Cambridge, United Kingdom, as part of a study on antibiotic resistance (see Table S1 at https://ndownloader.figshare.com/files/14983688). Five samples were from feces, and one was from blood. These were short-read libraries in a multiplex run on an Illumina HiSeq 2500 platform and were processed as previously outlined (43).

### High-molecular-weight DNA extraction.

Frozen stocks of the six isolates were streaked onto LB agar plates and grown overnight at 37°C. Single colonies were subcultured onto LB agar plates and incubated overnight at 37°C. DNA was extracted using a Lucigen MasterPure complete DNA and RNA purification kit. For each sample, a swab was used to sweep half a plate of pure colonies, which was suspended in 1× phosphate buffer solution (PBS). Samples were processed according to the manufacturer’s instructions, with elution in 70 μl of nuclease-free water. Pipetting was minimized to reduce shearing of the DNA prior to sequencing.

### Oxford Nanopore library preparation and sequencing.

DNA was quantified using a Quant-iT HS (high-sensitivity) kit (Invitrogen). DNA purity was checked using a NanoDrop (Thermo Fisher), and fragment size was confirmed by a Femto Pulse system (Nano Life Quest). The sequencing libraries were prepared using 1 µg DNA per sample and ligation sequencing kit 1D SQK-LSK109 with the barcoding extension kit EXP-NPB104 in accordance with the ONT protocols. The samples were combined using equimolar pooling and loaded onto a single 9.4.1 MIN-106 flow cell and sequenced on the GridION X5 platform under standard conditions.

### Illumina library preparation and sequencing.

The short reads used in this study were created as follows: bacterial genomic DNA was extracted using a QIAxtractor (Qiagen, Valencia, CA, USA) according to the manufacturer's instructions. Library preparation was conducted according to the Illumina protocol and sequenced (96-plex) on an Illumina HiSeq 2500 platform (Illumina, San Diego, CA, USA) using 100-bp paired-end reads.

### Oxford Nanopore base calling and adapter trimming.

The resulting fast5 read files (available at https://www.ncbi.nlm.nih.gov/sra/PRJEB30511 under accession numbers ERR3284704 to ERR3284709) were transferred to a separate Linux server 4.4.0 (Ubuntu 16.04.4) for analysis. Base calling was performed during the GridION run using ONT Guppy v0.5.1, and the resulting fast5 files from the initial run were converted to fastq format with Albacore v2.0 (ONT). The statistical data of the sequencing run was processed with MinIONQC v1.3.5 (44) based on the default Q score cutoff of 7. Adapters and chimeric reads were removed from fastq files using Porechop v0.2.4 (45) with demultiplex settings (see Fig. S1 at https://ndownloader.figshare.com/files/14983688). Standard outputs were saved as log files and then parsed. The quality of the final fastq files was assessed using FastQC v0.11.8 (https://www.bioinformatics.babraham.ac.uk/projects/fastqc/) and MultiQC v1.4 (46).

### Genome assembly and improvement.

We assembled the genomes using the conservative, normal, and bold modes of the long-read-only assembly pipeline in Unicycler v4.6. Previous work has suggested that Unicycler outperforms alternatives (21) that struggle to resolve plasmids (36). This workflow included the assembly polisher, Racon, which ran iteratively to minimize error rates of called bases (45). For comparison, short read-only and hybrid assemblies were also created using Unicycler v4.6. Briefly, during short-read-only assembly, Unicycler v4.6 employed SPAdes v3.12 to assemble short reads and then used Pilon v1.22 to polish the assembly. In hybrid assemblies, Unicycler v4.6 used Miniasm to piece together long reads first and applied SPAdes v3.12 to incorporate short reads and bridge gaps. Pilon was run 3 to 10 times for short-read assemblies and 5 to 10 times for hybrid ones, until no further changes were required to achieve the most contiguous and complete genome assemblies. The average numbers of changes by Pilon were 74.3, 100.2, and 125.3 for short-read assemblies and 234.5, 257.7, and 377.0 across conservative, normal, and bold modes, respectively.

### Genome assembly assessment and error rate quantification.

The quality of resulting assemblies was assessed using Quast 3.0 (47) according to the total assembly length, number of contigs, *N*_50_, GC content, and degree of replicon circularization. Assembly graphs were visualized with Bandage (48). The resulting contigs in each assembly were classified as chromosomal or plasmid using machine learning algorithms implemented in mlplasmids (22). Genome completeness was examined using the numbers of single-copy universal orthologous genes using BUSCO (Benchmarking Universal Single-Copy Orthologs) v3 with the gammaproteobacteria_odb9 database (49).

### Read depth estimation.

The read depth of each replicon was estimated by aligning the short Illumina reads and the long Oxford Nanopore reads to the completed genomes by use of Smalt v0.7.6 and BWA-MEM v0.7.17 (with the flag –x ont2d for ONT reads), respectively. SAMtools v1.7 was used to process the SAM files to BAM format, remove duplicates, and identify the coverage at each base of each assembly. The median value for each replicon was noted and was normalized using the median chromosomal depth of the same assembly.

### Genome annotation.

The genomes were annotated using Prokka v1.13.3 (50). *bla*_CTX-M_ alleles and their contexts were detected using the Multiple Antibiotic Resistance Annotator (MARA) (51) and by aligning the assemblies against the Comprehensive Antibiotic Resistance Database (CARD v3.0) to screen for matches with 100% identification only. Information on the detected AMR features and MGEs are retrieved from Galileo AMR (https://galileoamr.arcbio.com/mara/feature/list). Plasmid identification and typing were carried out using PlasmidFinder v2.0 (52). The plasmid-derived contigs from the assembled genomes were compared using BLAST v2.6.0 with a database of 10,892 complete plasmids (53). Their homology and annotation were visualized using EasyFig v2.2.2 (54).

### Phylogenetic analysis.

To provide a phylogenetic context for these six isolates, the Illumina read libraries of 63 published ST131 samples from references 8 and 56 from reference 31 were cleaned and trimmed using Fastp v0.12.3 (55), as were the short-read libraries of the six isolates from this study. These 125 libraries were *de novo* assembled with Unicycler v4.6 using NCTC13441 as a reference and annotated using Prokka. The 126 genomes were processed using Roary v3.11.2 (56) with a 95% BLAST v2.6.0 identity threshold to create a core genome alignment containing 4,457 SNPs using MAFFT v7.310 (57) spanning 3,250,343 bases and 3,350 genes of the NCTC13441 chromosome (a length similar to that described previously [20]). This core genome was used to construct a maximum likelihood phylogeny using RAxML v8.2.11 with the general time-reversible (GTR) model with gamma rate heterogeneity (58). Clade classification of the six isolates was based on a published ST131 phylogenetic analysis (8) with associated classification and *bla*_CTX-M_ allele data from references 8 and 31.

### Ethical approval.

The study protocol was approved by the National Research Ethics Service (reference 14/EE/1123) and the Cambridge University Hospitals NHS Foundation Trust Research and Development Department (reference A093285).

### Data availability.

Illumina reads were deposited under accession numbers ERR2138475, ERR2138200, ERR2138591, ERR1878196, ERR2137889, and ERR1878359 in the European Nucleotide Archive (ENA) under BioProjects PRJEB21499 and PRJEB19918. ONT reads were deposited under accession numbers ERR3284704, ERR328470, ERR3284706, ERR3284707, ERR3284708, and ERR3284709 (see https://www.ebi.ac.uk/ena/data/view/PRJEB30511 in the ENA or https://www.ncbi.nlm.nih.gov/sra/PRJEB30511 in the SRA under BioProject PRJEB30511; see also FigShare at https://doi.org/10.6084/m9.figshare.7554293.v1). Unicycler assemblies were deposited at FigShare (https://doi.org/10.6084/m9.figshare.7560458.v2).

## References

[1] Poolman JT, Wacker M 2016 Extraintestinal pathogenic *Escherichia coli*, a common human pathogen: challenges for vaccine development and progress in the field. J Infect Dis 213:6–13. doi:10.1093/infdis/jiv429. 26333944 PMC4676548

[2] Pitout JDD, DeVinney R 2017 *Escherichia coli* ST131: a multidrug-resistant clone primed for global domination. F1000Res 6:F1000 Faculty Rev-195. doi:10.12688/f1000research.10609.1. PMC5333602 28344773

[3] Goswami C, Fox S, Holden M, Connor M, Leanord A, Evans TJ 2018 Genetic analysis of invasive Escherichia coli in Scotland reveals determinants of healthcare-associated versus community-acquired infections. Microb Genom 4:e000190. doi:10.1099/mgen.0.000190. PMC6096937 29932391

[4] Ender PT, Gajanana D, Johnston B, Clabots C, Tamarkin FJ, Johnson JR 2009 Transmission of an extended-spectrum-beta-lactamase-producing *Escherichia coli* (sequence type ST131) strain between a father and daughter resulting in septic shock and emphysematous pyelonephritis. J Clin Microbiol 47:3780–3782. doi:10.1128/JCM.01361-09. 19741070 PMC2772609

[5] Van der Bij AK, Peirano G, Pitondo-Silva A, Pitout JD 2012 The presence of genes encoding for different virulence factors in clonally related *Escherichia coli* that produce CTX-Ms. Diagn Microbiol Infect Dis 72:297–302. doi:10.1016/j.diagmicrobio.2011.12.011. 22300954

[6] Calhau V, Ribeiro G, Mendonça N, Da Silva GJ 2013 Prevalent combination of virulence and plasmidic-encoded resistance in ST131 *Escherichia coli* strains. Virulence 4:726–729. doi:10.4161/viru.26552. 24128612 PMC3925704

[7] Totsika M, Beatson SA, Sarkar S, Phan MD, Petty NK, Bachmann N, Szubert M, Sidjabat HE, Paterson DL, Upton M, Schembri MA 2011 Insights into a multidrug resistant *Escherichia coli* pathogen of the globally disseminated ST131 lineage: genome analysis and virulence mechanisms. PLoS One 6:e26578. doi:10.1371/journal.pone.0026578. 22053197 PMC3203889

[8] Ben Zakour NL, Alsheikh-Hussain AS, Ashcroft MM, Khanh Nhu NT, Roberts LW, Stanton-Cook M, Schembri MA, Beatson SA 2016 Sequential acquisition of virulence and fluoroquinolone resistance has shaped the evolution of *Escherichia coli* ST131. mBio 7:e00347. doi:10.1128/mBio.00347-16. 27118589 PMC4850260

[9] Forde BM, Phan MD, Gawthorne JA, Ashcroft MM, Stanton-Cook M, Sarkar S, Peters KM, Chan KG, Chong TM, Yin WF, Upton M, Schembri MA, Beatson SA 2015 Lineage-specific methyltransferases define the methylome of the globally disseminated *Escherichia coli* ST131 clone. mBio 6:e01602. doi:10.1128/mBio.01602-15. 26578678 PMC4659465

[10] Johnson JR, Johnston B, Clabots C, Kuskowski MA, Castanheira M 2010 *Escherichia coli* sequence type ST131 as the major cause of serious multidrug-resistant *E. coli* infections in the United States. Clin Infect Dis 51:286–294. doi:10.1086/653932. 20572763

[11] Juhas M, van der Meer JR, Gaillard M, Harding RM, Hood DW, Crook DW 2009 Genomic islands: tools of bacterial horizontal gene transfer and evolution. FEMS Microbiol Rev 33:376–393. doi:10.1111/j.1574-6976.2008.00136.x. 19178566 PMC2704930

[12] Frost LS, Leplae R, Summers AO, Toussaint A 2005 Mobile genetic elements: the agents of open source evolution. Nat Rev Microbiol 3:722–732. doi:10.1038/nrmicro1235. 16138100

[13] Hinnebusch J, Tilly K 1993 Linear plasmids and chromosomes in bacteria. Mol Microbiol 10:917–922. doi:10.1111/j.1365-2958.1993.tb00963.x. 7934868

[14] Woodford N, Carattoli A, Karisik E, Underwood A, Ellington MJ, Livermore DM 2009 Complete nucleotide sequences of plasmids pEK204, pEK499, and pEK516, encoding CTX-M enzymes in three major *Escherichia coli* lineages from the United Kingdom, all belonging to the international O25:H4-ST131 clone. Antimicrob Agents Chemother 53:4472–4482. doi:10.1128/AAC.00688-09. 19687243 PMC2764225

[15] Nicolas-Chanoine MH, Bertrand X, Madec JY 2014 *Escherichia coli* ST131, an intriguing clonal group. Clin Microbiol Rev 27:543–574. doi:10.1128/CMR.00125-13. 24982321 PMC4135899

[16] Phan MD, Forde BM, Peters KM, Sarkar S, Hancock S, Stanton-Cook M, Ben Zakour NL, Upton M, Beatson SA, Schembri MA 2015 Molecular characterization of a multidrug resistance IncF plasmid from the globally disseminated *Escherichia coli* ST131 clone. PLoS One 10:e0122369. doi:10.1371/journal.pone.0122369. 25875675 PMC4398462

[17] Harrison E, Brockhurst MA 2012 Plasmid-mediated horizontal gene transfer is a coevolutionary process. Trends Microbiol 20:262–267. doi:10.1016/j.tim.2012.04.003. 22564249

[18] MacLean RC, San Millan A 2015 Microbial evolution: towards resolving the plasmid paradox. Curr Biol 25:R764–7. doi:10.1016/j.cub.2015.07.006. 26325139

[19] Shintani M, Sanchez ZK, Kimbara K 2015 Genomics of microbial plasmids: classification and identification based on replication and transfer systems and host taxonomy. Front Microbiol 6:242. . doi:10.3389/fmicb.2015.00242. 25873913 PMC4379921

[20] McNally A, Oren Y, Kelly D, Pascoe B, Dunn S, Sreecharan T, Vehkala M, Välimäki N, Prentice MB, Ashour A, Avram O, Pupko T, Dobrindt U, Literak I, Guenther S, Schaufler K, Wieler LH, Zhiyong Z, Sheppard SK, McInerney JO, Corander J 2016 Combined analysis of variation in core, accessory and regulatory genome regions provides a super-resolution view into the evolution of bacterial populations. PLoS Genet 12:e1006280. doi:10.1371/journal.pgen.1006280. 27618184 PMC5019451

[21] Wick RR, Judd LM, Gorrie CL, Holt KE 2017 Completing bacterial genome assemblies with multiplex MinION sequencing. Microb Genom 3:e000132. doi:10.1099/mgen.0.000132. 29177090 PMC5695209

[22] Arredondo-Alonso S, Rogers MRC, Braat JC, Verschuuren TD, Top J, Corander J, Willems RJL, Schürch AC 2018 mlplasmids: a user-friendly tool to predict plasmid- and chromosome-derived sequences for single species. Microb Genom 4:e000224. doi:10.1099/mgen.0.000224. PMC6321875 30383524

[23] Judge K, Hunt M, Reuter S, Tracey A, Quail MA, Parkhill J, Peacock SJ 2016 Comparison of bacterial genome assembly software for MinION data and their applicability to medical microbiology. Microb Genom 2:e000085. doi:10.1099/mgen.0.000085. 28348876 PMC5320651

[24] Leggett RM, Clark MD 2017 A world of opportunities with nanopore sequencing. J Exp Bot 68:5419–5429. doi:10.1093/jxb/erx289. 28992056

[25] Roer L, Overballe-Petersen S, Hansen F, Johannesen TB, Stegger M, Bortolaia V, Leekitcharoenphon P, Korsgaard HB, Seyfarth AM, Mossong J, Wattiau P, Boland C, Hansen DS, Hasman H, Hammerum AM, Hendriksen RS 2019 ST131 fimH22 *Escherichia coli* isolate with a blaCMY-2/IncI1/ST12 plasmid obtained from a patient with bloodstream infection: highly similar to *E. coli* isolates of broiler origin. J Antimicrob Chemother 74:557–560. doi:10.1093/jac/dky484. 30496481

[26] Goldstein S, Beka L, Graf J, Klassen J 2018 Evaluation of strategies for the assembly of diverse bacterial genomes using MinION long-read sequencing. BioRxiv 10.1101/362673. PMC6325685 30626323

[27] Partridge SR, Kwong SM, Firth N, Jensen SO 2018 Mobile genetic elements associated with antimicrobial resistance. Clin Microbiol Rev 31:e00088-17. doi:10.1128/CMR.00088-17. 30068738 PMC6148190

[28] Lartigue MF, Poirel L, Aubert D, Nordmann P 2006 In vitro analysis of IS*Ecp1B*-mediated mobilization of naturally occurring beta-lactamase gene blaCTX-M of *Kluyvera ascorbata*. Antimicrob Agents Chemother 50:1282–1286. doi:10.1128/AAC.50.4.1282-1286.2006. 16569841 PMC1426957

[29] Barlow M, Reik RA, Jacobs SD, Medina M, Meyer MP, McGowan JE Jr, Tenover FC 2008 High rate of mobilization for blaCTX-Ms. Emerg Infect Dis 14:423–428. doi:10.3201/eid1403.070405. 18325257 PMC2570810

[30] Petty NK, Ben Zakour NL, Stanton-Cook M, Skippington E, Totsika M, Forde BM, Phan MD, Gomes Moriel D, Peters KM, Davies M, Rogers BA, Dougan G, Rodriguez-Baño J, Pascual A, Pitout JD, Upton M, Paterson DL, Walsh TR, Schembri MA, Beatson SA 2014 Global dissemination of a multidrug resistant Escherichia coli clone. Proc Natl Acad Sci U S A 111:5694–5699. doi:10.1073/pnas.1322678111. 24706808 PMC3992628

[31] Matsumura Y, Pitout JDD, Peirano G, DeVinney R, Noguchi T, Yamamoto M, Gomi R, Matsuda T, Nakano S, Nagao M, Tanaka M, Ichiyama S 2017 Rapid identification of different Escherichia coli sequence type 131 clades. Antimicrob Agents Chemother 61:e00179-17. doi:10.1128/AAC.00179-17. 28584160 PMC5527616

[32] Lemon JK, Khil PP, Frank KM, Dekker JP 2017 Rapid nanopore sequencing of plasmids and resistance gene detection in clinical isolates. J Clin Microbiol 55:3530–3543. doi:10.1128/JCM.01069-17. 29021151 PMC5703817

[33] Stoesser N, Batty EM, Eyre DW, Morgan M, Wyllie DH, Del Ojo Elias C, Johnson JR, Walker AS, Peto TEA, Crook DW 2013 Predicting antimicrobial susceptibilities for *Escherichia coli* and *Klebsiella pneumoniae* isolates using whole genomic sequence data. J Antimicrob Chemother 68:2234–2244. doi:10.1093/jac/dkt180. 23722448 PMC3772739

[34] Branger C, Ledda A, Billard-Pomares T, Doublet B, Fouteau S, Barbe V, Roche D, Cruveiller S, Médigue C, Castellanos M, Decré D, Drieux-Rouze L, Clermont O, Glodt J, Tenaillon O, Cloeckaert A, Arlet G, Denamur E 2018 Extended-spectrum β-lactamase-encoding genes are spreading on a wide range of *Escherichia coli* plasmids existing prior to the use of third-generation cephalosporins. Microb Genom 4:e000203. doi:10.1099/mgen.0.000203. PMC6202452 30080134

[35] Moradigaravand D, Palm M, Farewell A, Mustonen V, Warringer J, Parts L 2018 Prediction of antibiotic resistance in Escherichia coli from large-scale pan-genome data. PLoS Comput Biol 14:e1006258. doi:10.1371/journal.pcbi.1006258. 30550564 PMC6310291

[36] George S, Pankhurst L, Hubbard A, Votintseva A, Stoesser N, Sheppard AE, Mathers A, Norris R, Navickaite I, Eaton C, Iqbal Z, Crook DW, Phan HTT 2017 Resolving plasmid structures in Enterobacteriaceae using the MinION nanopore sequencer: assessment of MinION and MinION/Illumina hybrid data assembly approaches. Microb Genom 2017:1–8. doi:10.1099/mgen.0.000118. PMC5610714 29026658

[37] Greig DR, Dallman TJ, Hopkins KL, Jenkins C 2018 MinION nanopore sequencing identifies the position and structure of bacterial antibiotic resistance determinants in a multidrug-resistant strain of enteroaggregative *Escherichia coli*. Microb Genom 4:e000213. doi:10.1099/mgen.0.000213. PMC6249433 30235111

[38] Wang Y, Yang Q, Wang Z 2014 The evolution of nanopore sequencing. Front Genet 5:449. doi:10.3389/fgene.2014.00449. 25610451 PMC4285804

[39] Tamma PD, Fan Y, Bergman Y, Pertea G, Kazmi A, Lewis S, Carroll KC, Schatz MC, Timp W, Simner P 2018 Rapid optimization of antibiotic therapy for multidrug-resistant gram-negative infections using nanopore whole genome sequencing. Available at SSRN: https://ssrn.com/abstract=3219539.

[40] Tyson GH, McDermott PF, Li C, Chen Y, Tadesse DA, Mukherjee S, Bodeis-Jones S, Kabera C, Gaines SA, Loneragan GH, Edrington TS, Torrence M, Harhay DM, Zhao S 2015 WGS accurately predicts antimicrobial resistance in *Escherichia coli*. J Antimicrob Chemother 70:2763–2769. doi:10.1093/jac/dkv186. 26142410 PMC11606221

[41] Su M, Satola SW, Read TD 2019 Genome-based prediction of bacterial antibiotic resistance. J Clin Microbiol 57:e01405-18. doi:10.1128/JCM.01405-18. 30381421 PMC6425178

[42] Lutgring JD, Zhu W, de Man TJB, Avillan JJ, Anderson KF, Lonsway DR, Rowe LA, Batra D, Rasheed JK, Limbago BM 2018 Phenotypic and genotypic characterization of Enterobacteriaceae producing oxacillinase-48-like carbapenemases, United States. Emerg Infect Dis 24:700–709. doi:10.3201/eid2404.171377. 29553324 PMC5875285

[43] Ludden C, Reuter S, Judge K, Gouliouris T, Blane B, Coll F, Naydenova P, Hunt M, Tracey A, Hopkins KL, Brown NM, Woodford N, Parkhill J, Peacock SJ 2017 Sharing of carbapenemase-encoding plasmids between *Enterobacteriaceae* in UK sewage uncovered by MinION sequencing. Microb Genom 3:e000114. doi:10.1099/mgen.0.000114. 29026655 PMC5605956

[44] Lanfear R, Schalamun M, Kainer D, Wang W, Schwessinger B 2019 MinIONQC: fast and simple quality control for MinION sequencing data. Bioinformatics 35:523–525. doi:10.1093/bioinformatics/bty654. 30052755 PMC6361240

[45] Wick RR, Judd LM, Gorrie CL, Holt KE 2017 Unicycler: resolving bacterial genome assemblies from short and long sequencing reads. PLoS Comput Biol 13:e1005595. doi:10.1371/journal.pcbi.1005595. 28594827 PMC5481147

[46] Ewels P, Magnusson M, Lundin S, Käller M 2016 MultiQC: summarize analysis results for multiple tools and samples in a single report. Bioinformatics 32:3047–3048. doi:10.1093/bioinformatics/btw354. 27312411 PMC5039924

[47] Gurevich A, Saveliev V, Vyahhi N, Tesler G 2013 QUAST: quality assessment tool for genome assemblies. Bioinformatics 29:1072–1075. doi:10.1093/bioinformatics/btt086. 23422339 PMC3624806

[48] Wick RR, Schultz MB, Zobel J, Holt KE 2015 Bandage: interactive visualization of de novo genome assemblies. Bioinformatics 31:3350–3352. doi:10.1093/bioinformatics/btv383. 26099265 PMC4595904

[49] Waterhouse RM, Seppey M, Simão FA, Manni M, Ioannidis P, Klioutchnikov G, Kriventseva EV, Zdobnov EM 2017 BUSCO applications from quality assessments to gene prediction and phylogenomics. Mol Biol Evol 35:543–548. doi:10.1093/molbev/msx319. PMC5850278 29220515

[50] Seemann T 2014 Prokka: rapid prokaryotic genome annotation. Bioinformatics 30:2068–2069. doi:10.1093/bioinformatics/btu153. 24642063

[51] Partridge SR, Tsafnat G 2018 Automated annotation of mobile antibiotic resistance in gram-negative bacteria: the Multiple Antibiotic Resistance Annotator (MARA) and database. J Antimicrob Chemother 73:883–890. doi:10.1093/jac/dkx513. 29373760

[52] Carattoli A, Zankari E, García-Fernández A, Voldby Larsen M, Lund O, Villa L, Møller Aarestrup F, Hasman H 2014 In silico detection and typing of plasmids using PlasmidFinder and plasmid multilocus sequence typing. Antimicrob Agents Chemother 58:3895–3903. doi:10.1128/AAC.02412-14. 24777092 PMC4068535

[53] Brooks L, Kaze M, Sistrom M 2019 A curated, comprehensive database of plasmid sequences. Microbiol Resour Announc 8:e01325-18. doi:10.1128/MRA.01325-18. 30637385 PMC6318356

[54] Sullivan MJ, Petty NK, Beatson SA 2011 Easyfig: a genome comparison visualizer. Bioinformatics 27:1009–1010. doi:10.1093/bioinformatics/btr039. 21278367 PMC3065679

[55] Chen S, Zhou Y, Chen Y, Gu J 2018 Fastp: an ultra-fast all-in-one FASTQ preprocessor. Bioinformatics 34:i884–i890. doi:10.1093/bioinformatics/bty560. 30423086 PMC6129281

[56] Page AJ, Cummins CA, Hunt M, Wong VK, Reuter S, Holden MT, Fookes M, Falush D, Keane JA, Parkhill J 2015 Roary: rapid large-scale prokaryote pan genome analysis. Bioinformatics 31:3691–3693. doi:10.1093/bioinformatics/btv421. 26198102 PMC4817141

[57] Katoh K, Standley DM 2013 MAFFT multiple sequence alignment software version 7: improvements in performance and usability. Mol Biol Evol 30:772–780. doi:10.1093/molbev/mst010. 23329690 PMC3603318

[58] Stamatakis A 2014 RAxML version 8: a tool for phylogenetic analysis and post-analysis of large phylogenies. Bioinformatics 30:1312–1313. doi:10.1093/bioinformatics/btu033. 24451623 PMC3998144

